# On the Affections of the Skin Induced by Temperature Variations in Cold Weather

**Published:** 1885-03

**Authors:** James Nevins Hyde

**Affiliations:** Professor of Skin and Venereal Diseases, Rush Medical College, Chicago


THE CHICAGO

MEDICAL JOURNAL AND EXAMINER.

VOL. L. MARCH, 1885. NO. 3.

### ORIGINAL COMMUNICATIONS.

### ARTICLE I.

ON THE AFFECTIONS OF THE SKIN INDUCED BY TEMPERATURE VARIATIONS IN COLD WEATHER. *By* JAMES NEVINS HYDE, M. D. *Professor of Skin and Venereal Diseases, Rush Medical College, Chicago.*

The consideration of the subject which is somewhat indefinitely announced in the title of this paper, possesses for the inhabitants of this part of the country, some importance. The subject involves a study of the changes induced in the skin of many persons subjected to the vicissitudes of the winter weather in the northwestern part of the United States; and its importance is derived from several considerations. Among the latter may be named the noteworthy difference in the behavior of the skin of different individuals thus exposed; the fact that this variability is in a minor degree subject to influences quite distinct from atmospheric changes; and last, but not least, though in part resulting from the reasons just stated, the fact that many persons are quite ignorant of the chief etiological factor in the production of these diseases, some even going so far as to deny with positiveness its causative operation.

A very moderate clinical experience establishes the fact that the change from a relatively high to a relatively low temperature, less often the reverse, is more efficient in the production of these skin disorders than a temperature which is for some time uniformly registered at a low point. Briefly and figuratively it may be said that it is not so much the shrinkage of the mercury to a low point, as its rapid play from one point to another that begets the mischief. It is unquestionably true, that the skin of many persons sympathizes with the summer variations of temperature, as, for example, that from 60° to 80° F., or from 70° to 90° F., which may often be recorded in this latitude in twelve hours of time. But it must not be forgotten that the winter changes are fully as abrupt, and, though possibly less noticed, are, as regards their influence upon the skin, probably far more effective. Here in the winter even greater variations occur, as from twenty degrees below zero to twenty above.

In the skin, which is the subject of the changes about to be described, the dip of the mercury in the thermometric tube may be held to be a rough index of the degree of aggravation of the cutaneous malady; but it would be fallacious to conclude that the sole etiological factor in each case was the coldness of the air alone. Patients who remain, during one of these periods of increasingly cold weather, in an apartment uniformly heated to a perfectly comfortable temperature, suffer, naturally, to a less extent than others, but do none the less suffer from the change in the atmospheric condition. Allowance should be made for the fact that the head, and often the shoulders, hands, and arms of one who sleeps in a warmed bed-chamber, are exposed somewhat at night to the lowered temperatures that are usually attained before the fires are radiating a greater supply of heat in the morning, but more must be admitted. There is something more efficient than even the transitory exposures to the cold air experienced in the opening of doors and windows. Thus the skin of certain infants, once affected by the disorders under consideration, who occupy chambers that are day and night kept by steam or other artificial heat at a fixed and perfectly comfortable temperature, will resent the change of condition in the atmosphere outside. What magneto-electric, chemical, or other modifications, may accompany the rapid transference of a great quantity of heat to its latent state upon the surface of the earth, need not be particularly discussed here. It is quite possible, even allowing for these, that a great retardation of the mode of motion we call heat, may necessarily involve the retardation or acceleration of other modes of motion, whose waves we may be at some time able to measure, and whose force to estimate in pounds.

Given one thousand human skins exposed to these influences, and, as we might readily understand from *a priori* reasoning, there will be in many, perhaps even a majority of them, no pathological results. This goes without saying. 'Tis the same when an equal number of persons swallow an indigestible aliment, of whom only a few suffer from diarrhœa. Even in the severest epidemics of yellow fever, thousands of the unacclimated escape. Unfortunately, in the present case, the proportion of those who suffer to those who enjoy immunity, cannot be statistically stated.

Naturally, those who suffer, suffer in different degrees. Here is a wide field opened.

The milder forms of these affections are familiar to all.

Almost every one recognizes the condition and appearance of the "chapped" hands, of the roughened faces, of the "frost-bitten" lips, nose, cheeks, and ears, and of the "chilblains" that affect the feet and hands. As the parts named are, to a greater extent than others, exposed either to the action of the weather or to objects chilled by this exposure, there is little or no difficulty in persuading the victims of these accidents as to the precise cause of their trouble. The difficulty, and this is often well-nigh insurmountable, arises when the action of cold air extends beyond the points of exposure, to the general surface of the body covered by the clothing. If the sturdy English of the last century had coined similarly homely phrases for "chaps" and "chilblains" of the skin covered by the clothing, there would have been far less reason for this present writing.

One of the commonest expressions of the resentment of the general surface of the skin is a decided cutaneous pruritus. Dr. Duhring, of Philadelphia, was first to describe this condition under the title, Pruritus Hiemalis, and the affection, as named by him, has received proper recognition by several foreign writers. It may be doubted, however, whether in the British Isles and upon the continent of Europe, whose climate is so happily modified by the gulf stream, the disorder is to be observed in anything like the severity and to the extent known here. Several circumstances, to which reference is made later in these pages, lead me to the conclusion that the region known as the Northwest, is occupied with a population which suffers from this and similar affections far more than do the dwellers upon either the eastern or western sea-board of this country.

The skin, which becomes the seat of this affection, is either gradually or suddenly involved, in persons of both sexes and all ages, and this independently of the previous habits of the sufferer as respects the bath. The cutaneous surface may be harsh, dry, rough to the touch, even exhibiting an exaggerated picture of the condition known as "goose-flesh," or be perfectly natural in appearance, soft, smooth, supple, and the seat of all normal secretions. The subjective sensations experienced in it may be first noticed in the cool weather of the autumn, or in mid-winter, or even during the relatively mild weather of the early spring, after the severity of the colder season has abated.

This subjective sensation is an itching, tingling, pricking, or burning, varying from the mildest and localized grades to the severest, affecting the entire bodily surface. Like most of the affections attended by cutaneous pruritus, the sensation of distress is decidedly aggravated after retiring to the bed at night, and this because of, first, the fatigue of the nervous system, which is usually greater at that hour; second, the change of temperature at the surface of the body necessitated by the change of clothing preparatory to occupation of the bed; third, the opportunities for scratching afforded by that change; and fourth, the increased vascular tone of the skin when re-heated within the bed-clothing. Another and usually milder exacerbation occurs on rising in the morning. During the day, when the attention of the nervous system is distracted by social and business engagements, and when the bodily clothing preserves the temperature of the surface at a relatively uniform point, there is less distress. A sudden or unguarded exposure, however, to the sources of heat or cold during the hours of the day, will often exceedingly annoy the sufferer. Thus, the physician who removes the clothing of his patient in order to inspect the covered surface of the body, will often find that he has in this way provoked an access of pruritus.

In all grades of severity, the disorder is either completely relieved or greatly mitigated by the advent of milder weather, or by removal to a more temperate climate. The tormented skin will often, however, in this latitude retain the souvenirs of its accidents till late in the spring.

The next picture presented clinically in this affection of the skin is a simple sequence of the pruritus described above. It results solely from the scratching practiced in order to relieve the itching. Here the skin is rubbed, torn, or abraded by the finger-nails or articles employed to supplant these, and becomes in varying degree the seat of resulting inflammatory changes. Pin-head-sized and larger reddened papules usually discrete, often aggregated in patches, occasionally widely separated, their apices torn or capped with a minute blood-crust, spring from a sound, a reddened, or an infiltrated integument. The streaks left upon the skin by the finger-nails are recognized as two, three, or four radii of linear abrasions, corresponding in number to the number of fingers used, either parallel, as when the hand is drawn like a rake over the skin, or, as Hebra pointed out, converging, when the thumb is rested upon the skin and made the *point d'appui* for the fingers approximated to it with such mischievous intent. Pushed to a still greater extent, this traumatism induces a severe artificial dermatitis or eczema, the skin becoming infiltrated, angry, reddened, excoriated, and fissured. Vesicles, pustules, and papules are seen, not often, however, intermingled, and the resulting crusts may be conspicuous, commonly having the superficial character and light tints of desiccated serum.

Now, it is a curious fact, that many patients in this condition are well aware of the fact that they have produced all the eruptive lesions themselves, and that the pruritus was the *fons et origo* of the whole disorder. A single well-directed question to this class of sufferers will at once throw a flood of light upon the diagnosis. But it is a much more singular fact that few, indeed, volunteer this statement. This is one of those puzzles that human nature, when subjected to critical examination, is constantly presenting to the physician. I cannot, as I write these lines, recall, in my entire experience, more than two or three instances when this precious bit of knowledge was given to me unasked. Many, it is true, are ignorant of the real state of the case. But whether informed or not of the fact, no one need depend upon question and answer for arriving at the proper conclusion. An inspection of the patient will furnish even more precise knowledge on this point.

On careful examination it will be seen that the lesions of the cutaneous surface exist either solely or in great preponderance upon the parts of the body most accessible to the hands. These are, in the order of frequency and for reasons that need not be named, first and prominently always the inside of the thighs; next, the outer faces of the buttocks, the upper and anterior portions of the legs, the popliteal spaces, the forearms and arms, the genital region in males, more particularly the front of the scrotum and the skin of the penis, the lower portion of the belly, and the infra-clavicular regions. Only the most determined and persistent scratcher attacks the interscapular space, below the scapular spines. Here, if anywhere, the skin will be seen either, as is almost the invariable rule, entirely destitute of lesions, or exhibiting a few scarcely irritated papules. Perhaps comparison will furnish better results. Thus, in the most severe cases of pruritus, the calves will suffer less than the tibial regions; the axillæ less than the groins; the small of the back less than the lower belly. It is a pure question of accessibility, and the story is writ large on the face of the body.

It is an unfortunate appanage of many skin disorders that they are liable to become greatly aggravated by the mischief which they indirectly determine. Thus, the man who strains in an effort to expel his engorged prostate from his rectum, and the woman who cannot avoid the attempt to swallow her swollen tonsil, commonly add to the acute inflammatory condition of these glands. A similar chain of events can be traced here. The pruritic skin, at last, not only itches far more in consequence of the traumatism inflicted upon it, but the traumatisms themselves, with the dermatitis they awaken, are more weather-sensitive than at the outset of the trouble.

Another form of cutaneous disorder under the influence of the atmospheric changes here considered is an eczema, usually of the papular type, occurring primarily, without marked precedent pruritus and independently of traumatism. Upon this point it is needful to speak with some caution. For there are patients who affirm that they have not scratched or otherwise wounded the skin, who have really produced this result in the unconsciousness of sleep; and, as Kaposi has well shown, the skin which is at one point the seat of an exudative affection is often so sensitive that the irritation occurring at this point may be the sole exciting cause of similar trouble at a distant point. Still, making allowance for all this, there are some patients who actually exhibit lesions due solely to the effects of climate.

As indicated above, the lesions are commonly pin-head-sized papules, reddened in various shades, the color being modified by the natural tint of the skin in different persons. They may be aggregated in patches, but are much more often widely dispersed and irregularly distributed over the surface. They are, as a rule, not most abundant in the several regions of the body already enumerated, unless the intervention of scratching has altered the natural features of the disorder. They may be associated with equally minute vesicles, or, more particularly in regions where the skin is delicate in structure, be in part transformed into such serum-containing lesions. Thus it is not rare to discover one or more of these papules capped with tiny vesicular apices at the roots of the fingers or in the interdigital spaces, the patient demonstrating to his physician the fact that they "contain water," by expressing a droplet of clear fluid from an isolated point. Similar lesions may often be found at the wrist, over the skin of the penis, and on the breast of women; and, when untorn, they are rather persistent, usually undergoing involution without forming a crust.

When the skin in this condition is subjected to scratching, the eruptive picture is very nearly that when the pruritic condition precedes the eruptive phenomena. But little difference between the two can be recognized in the case of dispensary patients, who exhibit, as a rule, the severer types of the disease. Patients of intelligence, observed in private practice, who carefully watch the integument and compare its condition one day with that of another, will often take great precautions to keep the hands from wounding the skin, and thus be able to point out with some exactness the evolution of individual lesions in some part of the body.

Other of the varieties of eczema may be similarly induced and similarly modified, less often, however, than that just noted. Pustular lesions are more often seen among the poorer class, when the surface of the skin has been long unwashed; and, as a result, the crusts they exhibit are more bulky and more deeply colored.

In the eczema of the cold season, not preceded by cutaneous pruritus, the character of the clothing worn plays some part in its production, though it is a minor one. The edge of the linen cuff and of the rough overcoat or cloak-sleeve playing at the wrists, evokes a plentiful crop of lesions at these points; and a similar result is obtained where the collars fret with their edge the neck. Woolen gloves during these seasons, are particularly prejudicial to the backs of the hands, where a really formidable eczema rubrum may develop. It must be admitted that the brilliant, aniline-dyed under-garments that are responsible for so much mischief in the warmer days of the summer when they are moistened with the sweat, are comparatively harmless when the mercury approaches the zero point. This must be due to the fact that in winter the skin is less often macerated with sweat, for day-laborers engaged in work that produces free prespiration in winter, exhibit the poisonous effects of these dyes. It may be said, in passing, that in order to set aside all possibilities of damage, these garments should be at all times interdicted to patients with cutaneous affections.

In yet other cases, the skin expresses its resentment of the atmospheric coldness, by a rebellious form of chronic urticaria. This is rather more common in women and children than in men. The wheals are evanescent, usually of small size, and sparsely or plentifully developed over the portions of the body covered by the clothing. In the warmth retained by bed-clothing, they become especially annoying. A common form is the papular wheal, sparsely scattered over the shoulders, about the waist, and over the arms, the lesions being often abraded by the nails in scratching. Care must be taken not to confound this condition with that in which the urtication is purely the result of such traumatism. When inspected in the hours of the day succeeding a night of distress from this source, one commonly finds on the skin of women who are thus tormented, only a few rosy or pinkish striæ or puncta, at the sites of the departed wheals. The natural appearance and condition of the integument in many of these cases, presents a striking contrast with the history, which is often detailed with the eloquence of feeling. Sleep during these accesses is quite forbidden; and the nervous system, as it suffers, contributes its share to the aggravation that is apt to follow. Exasperating recurrences are to be expected with each decided change in the temperature of the air.

There is another class of patients who suffer at these crises of cold weather with furuncles, which form about the buttocks, and in the skin and subcutaneous tissues of the thighs, neck, hands, arms, and legs. These are often multiple, but rarely formidable in size or depth of involvement of the tissues. They occur both within and beyond eczematous patches, and even in persons who have suffered from only the more trifling of the eruptive disorders enumerated above. Many patients in this category cannot be recognized as suffering from "the depressed state of the system" commonly assigned as the cause of furunculosis. They are often indeed in a condition of sound general health, the boils resulting solely from the cutaneous irritation.

Lastly, it is proper to remark, in passing, that the variations of atmospheric temperature in cold weather may exert an unfavorable influence upon several skin affections of widely diverse origin, even upon those dating from the summer season. Among them may be named, typical eczema capitis of infants with seborrhœic complications, and the psoriasis of adults. These and others by aggravation or recrudesence testify to the influence of the season. It is understood, of course, that there are many patients who suffer from cutaneous diseases that are greatly ameliorated in the winter months.

Having thus briefly summarized the conditions in which the skin may be placed by the action of temperature changes in cold weather, I desire in the following pages to discuss more particularly certain questions of diagnosis and etiology which are thus naturally suggested. These questions occur more frequently in the presence of pruritus hiemalis, papular eczema, and the changes induced in both by the traumatism of scratching. According to the view which they take of these questions, the most of patients and too many physicians may be divided into two classes; the first, those who believe them to be due to parasites: the second, those who charge that they result from "impurities in the blood."

Touching the opinions held by the first class, it may be remarked that there are few counties in the Northwestern States that do not, popularly speaking, possess an itch called by a name of their own. Many are at one in contenting themselves with the widely accepted term, "prairie itch," which covers a great deal of ignorance and may be well relegated to a place by the side of the "army-itch," whose history did not survive the few years of civil war. Other names no less suggestive of a popular belief as to the origin of these troubles, have no better *raison d'être.* There are schools, and schools of belief in these questions. Many hold that the cause is a "microscopic animalcule," others, that the disease is really scabies, as a result of which, vain attempts are made for weeks at a time to relieve the eruptive disorder by inunctions of sulphur-containing salves. For five years past, I have been in receipt annually of a number of letters from intelligent physicians in Dakota, Nebraska, Minnesota, Iowa, and portions of Illinois, whose contents are so similar that they correspond to a remarkable degree. The writer usually announces that in the neighborhood where he is engaged in practice, a peculiar form of "itch" has appeared, affecting the greater number of all the inhabitants of the region, men, women and children; eminently contagious in character; and incurable by the ordinary methods of treatment. Some of these correspondents declare, that "every one has it." These letters are alike also in the fact that they commonly begin to appear in the late autumn or winter, and cease in the spring.

Of course it would be improper to decide positively on the diagnosis of cases of disease, without a personal and critical examination of the patient. But occasionally, a selected representative of this class of sufferers has been presented to me, in the person of some well-to-do farmer, anxious for relief and ready to incur the expense of a trip to the large cities in the hope of securing it. In every one of the cases presented, the sufferer has been found affected with some one of the disorders of the skin described above.

A few weeks ago, the following announcement appeared in the public press:

*The Itch.*—Louisville (Ky.) *Times:* Louisville has the itch. So have Jeffersonville and New Albany. It is probable that 5,000 people about the falls are daily applying antidotes for the old-fashioned malady remembered by the children of twenty years ago as the "itch." The remedy then was a nightly greasing from head to foot with hog's lard and sulphur. That is also the remedy now. The malady's introduction is credited to the recent influx of tramps from the North. The physicians estimate that every twentieth person in Louisville has it, and say they are kept busy in consultations and writing prescriptions, while the druggists are besieged by people in search of a cure.

The "tramps from the North," in this instance, were most probably the series of cold waves from the Manitoba region, which have lately been surpassing their usual limits and reaching with unwonted severity even over some of the Southern States.

While we certainly should not venture to diagnosticate diseases which we have never seen, we are not left without a basis of fact from which to reason about them. A few years ago, we had not, in this country, any such data. But now, the returns compiled by the Statistical Committee of the American Dermatological Association, furnish valuable testimony on such points as these. It has been objected to these returns that they claim to be what they are not, viz., statistics of all the skin diseases that occur throughout the country. No claim could be more preposterous. The very objection involves the one feature for which these statistics are really valuable. They are not gathered indiscriminately from all sources, but are solely obtained from careful records made by a few men at widely different points in the country, each of whom has special facilities for the observation of skin diseases, and a reputation for some skill in their diagnosis. Consequently, one chief objection to the mass of medical statistics in general, is here eliminated.

From the year 1878 to 1882, this committee reported 58,617 cases of skin diseases of all kinds; and the total number of cases of scabies included in the list was but 665, that is 1.10 per cent. The year 1884 was an exceptional one as regards scabies. Out of 9,329 cases of cutaneous diseases, reported by these gentlemen from Boston, New York, St. Louis, Chicago, and Canada, there were 339 cases of scabies. This relatively great increase was largely due to local causes, however; for of these cases, Boston reported 179, and accompanied these figures with a note calling attention to the increase. It is interesting to specify that of the cases collated in 1884, only 33 were seen in private practice, the remainder, 306, being observed in dispensary or public patients.

When we look to the several countries of Europe for information on this point, we find that Scotland enjoys an unenviable preëminence in the preponderance of itch. In 1873, Dr. Mc-

Call Anderson, of Glasgow,* classified ten thousand consecutive cases of skin disease occurring in the public practice of his city, and of these no less than 2,527 were cases of scabies. Of one thousand consecutive cases of skin disease occurring there in private practice, only 44 were classed as scabies.

In the year 1868, the late Sir Erasmus Wilson † collated the statistics of 5,000 consecutive cases of skin disease, as they occurred among the "wealthier classes" in Great Britain. Of this number, 184 only suffered from scabies.

Now, looking further into these facts, it is apparent that both in Scotland and America, these figures are derived from observations made in cities. It is in the large capitals of Europe that the poor and the filthy are closely congregated; furnish thus the best culture-field for acari; and from them, by annual emigration, the American colonies have been regularly recruited. It is for this reason that the seaboard cities of this continent are always able to report a somewhat larger proportion of cases of scabies than can the cities of the interior; and, as for the country towns in the interior, the ratio of from one to three per cent. of the same disorder to all cases of skin disease falling under the observation of physicians, must be greatly reduced.

Once it was different. In the old Colonial and Revolutionary days, our ancestors had come so lately from the country where the itch prevails, that the accidents of their former environment were retained to an uncomfortable extent. One can scarcely look over the small news sheets of that period without seeing several advertisements calling attention to some specific, vaunted for the itch. These notices, indeed, supplied the pabulum for the journalists of this day, that is now

---

* On the Treatment of Diseases of the Skin, 1873.

† Jour. of Cutaneous Medicine, vol. I., 1868, pp. 389.

derived from the enterprise of the men who sell "liver-pads" and "blood purifiers." Gradually the people of the country began, in a large sense, a life of independence; and in proportion as they did this, they ceased to suffer from the itch. The country was too large, water too abundant, soap too cheap, and big cities too few, to furnish sufficient encouragement to the *sarcoptes hominis* to linger here long. But when the year 1861 found the country embarrassed with a civil war, hundreds of thousands of its citizens were congregated in regiments, camps, prisons, barracks, and hospitals. Among them were many newly arrived immigrants, who enlisted almost as soon as they landed; and brought the itch once more into conditions so like those in which it flourishes in the overcrowded cities of Europe, that the acari again bred freely on this continent. When the war was ended, and the soldiers returned to their country homes, again the proportion of scabies to other cutaneous disorders gradually declined to the point represented by the figures given in the consolidated five-years-report of the statistical committee to which attention has been directed above. It is interesting to note that a similar proportionate increase and subsequent decrease in the number of cases of scabies recorded in England was observed after the return of the British army from the Crimea.

It follows, then, that if there are "5,000" persons in Louisville suffering from the itch, and thousands more in the cities of the Northwest, from which the reports come that "every man, woman, and child in the town" is affected with the same disease, it is one of the most exceptional and surprising facts in the medical history of this country, and quite unequaled by anything known to exist in the old world.

Still, and this is a matter of great moment, if there are even from one to three per cent. of all cutaneous diseases which depend upon the presence of acari, it is highly important that the symptoms of their presence and ravages be well understood. No man is so good a diagnostician as he who understands the exceptions to the rule. For that reason it will not be out of place to look for a moment into the features by which scabies is to be differentiated from the pruritus and eczemas that furnish the real theme of this paper.

They are all, indeed, surprisingly alike. The reason for this resemblance is clear. In all, the skin is greatly irritated ; in the one case, by cold air ; in the other, by a parasite attacking the skin. The result is the same, a pruritus differing in degree in different cases, but which may be as severe in one disease as in another. Then follows the scratching, which gives almost the same clinical portrait to each of the affections named. No wonder they resemble each other. No wonder that the physicians in Louisville are reported to be rubbing sulphur salves over their tormented patients. The plates illustrating scabies in Hebra's superb atlas will answer very well indeed for many typical cases of the diseases now prevalent in the Northwestern States. Hebra had in his life observed 40,000 cases of scabies, and drew a masterly portrait, which is to-day unrivaled, of that affection. But it may be doubted whether he had in all his experience encountered the skin diseases which most closely resemble the clinical picture he has drawn of scabies, for the climate in which he lived does not seem to have supplied them to his wards. It was reserved for an American, and after him an Englishman,* to first name and describe the disease as it occurs in ruder climates than that of Austria. We might, however, believe, after a perusal of their papers, that neither of these two eminent writers, for

* See Mr. Jonathan Hutchinson's paper on "Winter-Prurigo," Lectures on Clinical Surgery, vol. I, p. 100. London, 1878.

the same reason, appreciated, when they wrote, the extent and gravity of this group of disorders as they occur in the north-western part of this country in the season of winter.

Naturally, the first point to which we turn in the study of the differential diagnosis of scabies, is its contagiousness. One excellent author describes this disorder as "highly contagious;" and we know that it may be communicated by the shake of the hand. But we must make some reserve here. While scabies may be communicated by hand-shaking, it is rarely so communicated. I have again and again handled patients affected with the disease without incurring any unpleasant results in my own person; and it is a well-known fact that in the amphitheatre of medical schools and hospitals, patients with scabies are frequently passed around among physicians and students for close personal examination, with the rarest effect of spreading the disease in this way. There are several facts which must be considered in this connection. In the first place, the acarus family find nutriment, shelter, and all they require for comfort on the person of the individual whose skin they inhabit; and there is no great inducement for them to colonize upon a strange skin at the instant of the first opportunity offered. In the second place, the transfer of a male acarus alone, from one person to another, would not ensure a generation of the young. In the third place the unimpregnated female could not alone accomplish a large success as regards a progeny; and as for the impregnated female, Hebra on several occasions failed to induce scabies when one such only was transferred intentionally to a sound skin and seen to penetrate it. Lastly, the eggs alone would not suffice, for these have to be nicely planted within the epidermis, in order to be hatched safely to maturity. In brief, only the more intimate contacts of the bed at night, and the application of nails charged with acari of both sexes, especially the young, are to be regarded as most effective for the transmission of the disease. That is one reason why nearly seven men are found to bè affected with scabies to one woman. Women are, as a rule, more inclined to sleep alone, or with those only to whom they have family ties; while laborers, boys, apprentices, and persons of that class, including those who are strangers to each other, at times occupy the same beds, especially in the large cities where they are often huddled together at night like swine. Everything said and done, scabies is one of the filth diseases; while the winter disorders of the skin affect all classes alike; women as much as men, those who wash as much as those who do not wash, the occupants of the mansions of the wealthy and of the cottages of the poor. Indeed, when any disease affects equally all classes of society, its cause should always be sought among the more common rather than among the rare agencies of the generation of disease. Parasitic will probably be always numerically fewer than non-parasitic disorders. The general involvement of all or a very large number of people at one time in a given community, points always to a disease-cause of very wide operation; and such a cause, in the winter diseases under consideration, can be recognized in temperature changes in the atmosphere.

Nothing, however, could be more untrustworthy, than the testimony as to the origin of their disease, given by patients who have been subjected to the operation of that cause. They will often describe with astonishing minuteness the occasion of their infection, and the particular individual from whom they contracted their disease. In this way, the joint occupancy of a bed with a friend or a stranger; the apartment of a hotel in which they have been guests; the wearing of garments loaned them by their friends; and even more casual accidents of personal contact, are urged as the mode by which their disease was transmitted to them.

Looking, next, closely to the eruptive phenomena in scabies, it is needless to say that the burrow made by the female acarus as she penetrates the epidermis, is regarded as a pathognomonic symptom. But it should not be forgotten that the burrow may be wanting, or not discovered; first, because the acari actually upon the skin have not yet excavated these tunnels; or, second, because the fingers have torn the skin so as to disguise the lesions. In the first case, time will reveal a difference; and in the second, a more careful search must be made for the intruders.

When the burrow exists, it can be most perfectly recognized in the inter-digital spaces and on the skin of the penis, as a tangential line, running from a vesicle, papule, or pustule, to a distance of from one-eighth of an inch to an inch. It resembles a beaded, dotted, yellowish, or blackish thread, the color being more pronounced in comparison with a fresh colored and washed skin, and less marked in contrast with a soiled surface; being, in a soiled and subsequently washed integument, most conspicuous in proportion as the small puncta have served to entrap particles of dirt. The cuniculus may be curved, angular, or tortuous; and occasionally may be seen well nigh completely covered by a bulla, pustule, or vesicle extending its entire length. In such cases, however, the female always penetrates beyond the peripheral wall of such lesion, working her gallery beyond it and more deeply, lest she be lifted by the exudation out of reach of the succulent rete where she feeds.

The intruder may be recognized always at the terminal extremity of her gallery, for it is now known that she does not in her life-time leave it for any purpose, as was at one time taught. The female acarus here shows as a minute, whitish, clearly defined dot, presenting a contrast in this particular with the blackish fæces in the gallery behind; and may be, in a good light, by a person of some dexterity and fair eye-sight, extracted on the point of a cambric needle, from her lodging point. It is important to know that this parasite may be recognized by the unaided human eye; and its characteristic tortoise-like body exhibits most of its anatomical peculiarities under a glass enlarging the figure but one hundred diameters. It is not, therefore, a "microscopic animalcule, totally invisible to the eye," which is often claimed to be the cause of the disorders forming the theme of this paper, but is an object that was recognized by man before he ever looked through the first microscope. In the last century even, "old women" obtained a reputation for the discovery and extraction of the itch-mite from the human skin; and the many sharp-eyed practitioners in the Northwest, in the middle hours of even the shortest days of the winter, are surely able to do as much and more.

The "head" of the gallery is usually whitish, where the parasite first entered the skin; and is also more elevated than the "tail," where the acarus rests after laying its dozen or more of eggs. At times, the entire cuniculus forms an elevated ridge, rather than a thread-like depression, with white dots along its summit. When the roof of the vesicle at "the head" is torn off by scratching, the effect is to produce a reddened spot at its site, surrounded by a whitish moat running around the spot to the entrance of the gallery.

The itching which results from this epidermic tunnelling is severe, and noticeably more severe than would be suggested by the moderate number of skin lesions visible. When these lesions (puncta, vesicles, pustules, blebs, papules, resulting crusts, furrows, excoriations, etc.,) are found upon the hands, the itching becomes so great that the infested person scratches also the accessible parts of the skin, where there were originally no acari, such as the inside of the thighs, the lower belly, etc., as Hebra suggests, simply because they are "handy." Hence it is that the picture comes to resemble that of all pruritic and scratched skins. The regions affected by the eruption are the palms (especially of women and children) and dorsal surfaces of the hands; the sides and roots of the fingers, and toes; the wrists; the feet (and, especially in women, the delicate skin of the feet near the instep, partly dorsal, partly plantar in situation;) the buttocks, (more particularly in those who are seated in the trades and occupations of life); the extensor faces of the joints; the belly; the penis, in men; the anterior folds of the axillæ; the nipple and breast of women; and the elbows and knees, rather than the popliteal space and bend of the elbow. Hebra points to the fact that between two parallels, one drawn through the nipples, and another at a short distance above the knees, on the anterior face of the body, can be recognized the greater part of the eruptive lesions in every case of scabies, where the skin has been well scratched. He adds that pustules on the buttocks are almost conclusive evidences of the presence of the acarus in cobblers and workmen who sit at their trade; that pustules on the fingers and toes, and of the hands and feet, especially in children, are almost equally conclusive evidences of the malady; and that other regions pressed upon by clothing, such as those touched by trusses, pads, corsets, etc., are places where the parasite multiplies freely. The exemption of laundresses, persons employed in public baths, and those whose occupation requires much application of soap and water to the surface, has been noted by several authors. In infants, the parts brought into contact with the breast and hands of the mother and nurse, are those most apt to be involved; such as the face, the palms of the hands, buttocks, and, indeed, when infants are at the breast, all parts of the body.

Lastly, as regards the progress of the case, the winter disorders described above are better and worse according to the temperature changes in the atmosphere, and are not benefited to any appreciable extent by the external use of parasiticides; while the course of unrelieved scabies is ever toward a steady aggravation, being readily arrested with, at the worst, sequelæ of a mild eczema, when properly treated.

I have devoted so much space to the important question of the distinction between the winter diseases of this part of the country and scabies, that space is not left in which to do justice to the opinions held by the second class of patients described above. These believe that the maladies of the skin here discussed are due to "impurities of the blood." This is an old doctrine. It dates from the period of the "melancholic juices" of Galen, and suggests that aphorism of Hippocrates, in which it is declared that "when furfuraceous particles are discharged along with the thick urine, there is scabies of the bladder." It runs like a blood-red strand through almost every cord used since in medicine, wherewith to tie up a bundle of errors. Even the itch did not escape this misfortune, after it had been demonstrably produced by the presence of acari. One of the forlorn pictures presented by dermatology to the history of medicine is that representing the eminent Devergie, writing in 1877,* with a full knowledge of all the damage produced in the skin by the presence of the sarcoptes hominis, that he "attributes a secondary importance to the acarus in the itch," and that this malady can be "spontaneously induced." The internal remedies that have been administered for relief of the itch no man can number. Unfortunately,

* Traité Prat. des Maladies de la Peau, Paris, 1877, p. 565.

the "blood purifying" remedies employed in the winter diseases of the skin almost invariably aggravate the latter. The worst phases of these maladies are those in which the salts of potash, mercury, and arsenic have been pushed to the fullest extent, in the vain hope of thus securing relief.

The therapeutic problem presented is this: first, to allay the irritability of the skin; second, to relieve the eruptive symptoms, whether these be the result of traumatism or of the action upon the skin of the cold air; third, to accomplish these results when the major cause of the disease is periodically in full operation, since it is obvious that the number of sufferers is far too great to admit of their general removal to a more temperate climate; fourth, to accomplish these results without the use of anodynes and narcotics to induce sleep at night, the most of which in the end aggravate the disorders by their secondary effects upon the nervous system; and, lastly, to set aside the minor causes of aggravation of the disease, in a skin rendered unduly sensitive by the operation of the major cause described.

As these pages have already surpassed the limits originally intended, the consideration of this part of the subject must be deferred to a second paper.

